# Effects of dribbling restrictions in small-sided games on aerobic and anaerobic fitness in youth basketball players

**DOI:** 10.3389/fphys.2025.1550580

**Published:** 2025-01-31

**Authors:** Mingbang Li, Liang Tan, Hong Wu, Jianwei Wu

**Affiliations:** ^1^ College of Physical Education and Health, Geely University of China, Chengdu, China; ^2^ Hospital of Chengdu University of Traditional Chinese Medicine, Chengdu, China; ^3^ School of Wushu, Chengdu Sport University, Chengdu, China; ^4^ School of Sport and Health, Chengdu University of Traditional Chinese Medicine, Chengdu, China

**Keywords:** adolescent players, team sports training, physical conditioning, constrained training task, youth sports

## Abstract

**Introduction:**

Imposing constraints such as limiting dribbling in smallsided games (SSGs) is known to increase physiological and locomotor demands. However, the long-term effects on physical adaptations remain unexplored. This experimental study aimed to compare the impact of free-play SSGs (freeD) and limited-dribbling SSGs (limitedD) in SSGs on the aerobic and anaerobic adaptations of youth basketball players.

**Methods:**

Forty-five youth basketball players (aged 15.7 ± 0.6 years, with 4.2 ± 0.7 years of experience) were randomly assigned to two experimental groups (freeD and limitedD) and a control group (not exposed to SSG interventions). During the eight-week intervention, the experimental groups participated in additional SSG sessions twice a week, with session work time durations ranging from 12 to 16 min. Both experimental groups followed identical SSG formats, court dimensions, and training regimens, with the only difference being that one group participated in free play while the other group was prohibited from dribbling during progression. Aerobic capacity was assessed using the Yo-Yo Intermittent Recovery Test Level 1 (YYIRT), while the 30-second Wingate Test measured peak power output (PPO) and average power output (APO) at baseline and post-intervention. Statistical analysis was conducted using a mixed ANOVA to examine the interactions between time and group.

**Results:**

Comparisons of YYIRT between groups at post-intervention revealed that limitedD performed significantly better than both freeD (*p* = 0.035; *d* = 1.038) and the control group (*p* < 0.001; *d* = 2.050), while freeD also showed significantly better performance (*p* = 0.021; *d* = 0.082) than the control group. Regarding PPO, limitedD was significantly better than the control group (*p* = 0.043; *d* = 0.943). Finally, for APO, limitedD was significantly better than both freeD (*p* = 0.043; *d* = 0.928) and the control group (*p* < 0.001; *d* = 1.793), while freeD also exhibited significantly better performance than the control group (*p* = 0.046; *d* = 0.036).

**Conclusions:**

Limiting dribbling in basketball SSGs is more effective than free play. This makes it a potentially valuable strategy for designing SSGs in basketball training. Coaches may consider incorporating limited-dribbling conditions into SSGs to boost the intensity of training sessions, improve cardiovascular endurance, and enhance anaerobic power.

## 1 Introduction

In recent decades, ecological-based drills have become increasingly popular in team sports training (Davids et al., 2013). These drills integrate task constraints into the design and organization of games, helping to enhance players’ responses and better align their actions with the objectives of the task (Davids et al., 2013). Among these ecological-based drills, small-sided games (SSGs) have become particularly popular due to their ability to maintain key dynamics and characteristics of formal games, while integrating modifications such as smaller court dimensions, adjusted rules, or additional scoring targets to shape players’ behaviors according to coaching objectives (Arias et al., 2012; Conte et al., 2015; Mateus et al., 2019; Mateus et al., 2020).

Research in SSGs highlights their effectiveness for youth basketball training. They simulate real-game conditions while providing more opportunities for individual involvement (Jose Figueiredo de Souza et al., 2024). The reduced number of players increases ball interactions, enhancing the development of technical skills like dribbling, passing, and shooting (Klusemann et al., 2012; El-Gammal, 2016). Furthermore, the dynamic nature of SSGs promotes decision-making and tactical awareness by placing players in situations that require quick thinking and spatial adaptation (Bredt et al., 2020; Bredt et al., 2022; Camacho et al., 2021). Physiologically, SSGs also replicate the high-intensity intermittent efforts characteristic of basketball, with opportunities for improving cardiovascular fitness in young athletes (Delextrat and Martinez, 2014; Vaquera et al., 2018; Clemente et al., 2021).

Effective aerobic and anaerobic conditioning is vital for youth basketball players to meet the physical demands of the sport and support their overall development (Delextrat and Cohen, 2008; Delextrat and Martinez, 2014; Delextrat et al., 2018). Aerobic conditioning improves cardiovascular endurance by promoting adaptations such as increased stroke volume, capillary density, and mitochondrial efficiency (Wang and Wang, 2024). These adaptations enable players to maintain performance levels and recover effectively during breaks (Hoffman et al., 1999). Anaerobic conditioning, on the other hand, enhances the ability to produce energy through glycolysis, boosting muscle buffering capacity, phosphocreatine resynthesis, and lactate tolerance—essential for high-intensity actions (Zadro et al., 2011). SSGs offer an engaging and potentially effective approach to improving these physiological capacities due to their intermittent, high-intensity nature (Arslan et al., 2022). The frequent transitions between effort and recovery in SSGs stimulate both aerobic and anaerobic energy systems, fostering adaptations such as improved oxygen utilization, enhanced glycogen storage, and quicker recovery kinetics (Delextrat and Martinez, 2014; Delextrat et al., 2018).

Designing appropriate SSGs is crucial, as manipulating various task rules can elicit different physiological effects. For instance, Conte et al. (2016) demonstrated that varying the number of players and training regimes significantly influences the physiological and technical demands of basketball drills. Similarly, Mateus et al. (2019) found that increasing the number of scoring targets during simulated youth basketball games alters the physical demands placed on players. Reviews also support this idea, as it is well known that smaller formats (e.g., 1v1 or 2v2) significantly increase physiological demands, including heart rate, perceived exertion, and blood lactate concentrations, compared to larger formats (Clemente, 2016; Li et al., 2024). Additionally, larger courts (compared to smaller ones) may offer more opportunities to emphasize intensified locomotor demands, leading to increased running distances and higher running thresholds (Clemente, 2016; Li et al., 2024). However, there are other ways to influence physiological and locomotor demands in basketball, such as by modifying rules that target specific conditioning actions. For instance, altering dribbling rules in basketball SSGs can significantly impact the acute physiological and physical demands on players (Conte et al., 2015; Ferioli et al., 2020). For instance (Ferioli et al., 2020), found that limiting dribbling in 3v3 games resulted in a greater number of turnovers and passes, as well as increased time spent at higher locomotor intensities. Moreover (Conte et al., 2015), found that SSGs with no dribbling imposed greater physiological stress compared to free play.

These acute changes may influence long-term adaptations, although direct experimental evidence on this aspect is currently lacking. Consistently exposing players to higher movement intensities and increased workloads during training (such as limiting dribbling versus free play) could potentially enhance aerobic capacity and anaerobic performance over time as observed previously (Delextrat and Martinez, 2014; Delextrat et al., 2018). Given this research gap, our research aimed to compare the impact of free-play (freeD) and limited-dribbling (limitedD) in SSGs on the aerobic and anaerobic adaptations of youth basketball players. We hypothesize that limitedD may lead to greater physiological adaptations, as the acute physiological responses are generally more intense in this scenario (Conte et al., 2015; Ferioli et al., 2020).

## 2 Materials and methods

### 2.1 Participants

The G*Power software (version 3.1.9, Universität Düsseldorf, Germany) was used to calculate the sample size for the study. Based on an effect size f of 0.808, derived from a direct calculation of eta squared (0.395) identified in a previous study on the impact of SSG on aerobic performance (Delextrat et al., 2018), the calculation considered three groups and two measurement points. With a desired statistical power of 0.95 and a significance level of 0.05 for the ANOVA repeated measures within-between interaction, the recommended total sample size was 12 participants.

**FIGURE 1:**
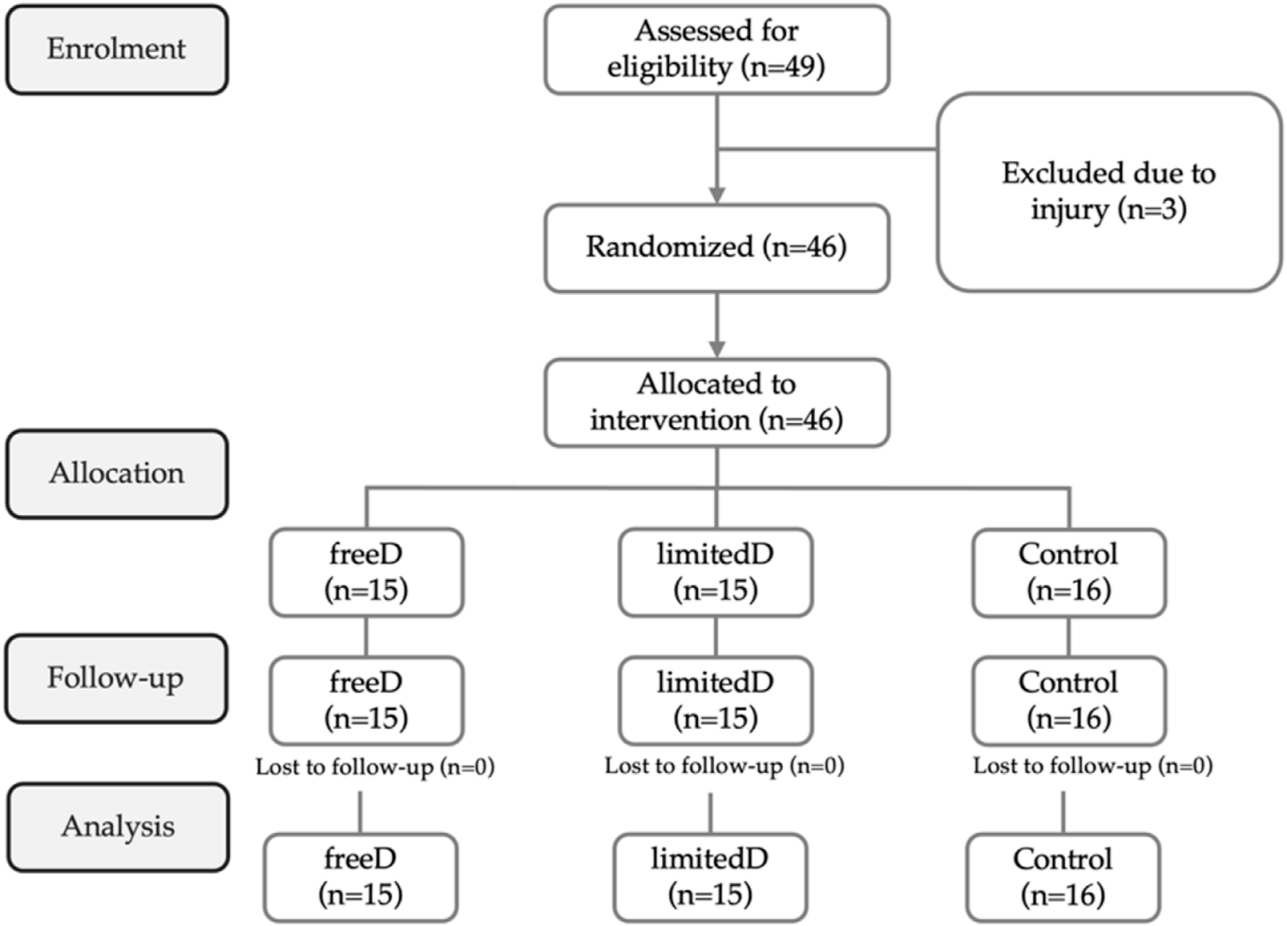
Participant flowchart. freeD: small-sided games in free play; limitedD: small-sided games with limited dribbling.

Following the recruitment process across five teams, 49 players volunteered for participation. However, three players were excluded due to missing the initial evaluation because of injury. As a result, 46 eligible players were randomly assigned to one of the three groups (Figure 1).

A total of 46 male youth basketball players (age: 15.7 ± 0.5 years; height: 175.7 ± 5.8 cm; body mass: 63.7 ± 7.4 kg; body mass index (BMI): 20.6 ± 1.7 kg/m^2^) classified as trained/developmental level according to the Participants Classification Framework (McKay et al., 2022), were enrolled in the study. The inclusion criteria for the study were as follows: (i) participation in both evaluation moments (before and after the intervention), (ii) a minimum of 2 years of basketball experience, (iii) attendance of at least 90% of regular training sessions, (iv) no injury or illness during the experiment, (v) no involvement in additional conditioning training programs, and (vi) male gender.

These players, competing at the regional level, participated in regular training sessions three times a week, focusing on competitive preparation. Each session lasted 85–110 min and was structured to improve both competitive readiness and skill specialization. It included a general warm-up, followed by specific physical conditioning, technical development and individual drills, positioning and strategy training, and concluded with a formal game and a cool-down phase.

For the specific groups, the freeD group (n = 15) had an average age of 15.7 ± 0.6 years, an average height of 172.5 ± 4.5 cm, an average body mass of 60.1 ± 6.2 kg, and an average BMI of 20.2 ± 1.6 kg/m^2^. The limitedD group (n = 15) had an average age of 15.7 ± 0.5 years, an average height of 178.3 ± 5.9 cm, an average body mass of 66.7 ± 7.1 kg, and an average BMI of 21.0 ± 1.9 kg/m^2^. The control group (n = 16) had an average age of 15.8 ± 0.5 years an average height of 176.4 ± 5.5 cm, an average body mass of 64.3 ± 7.9 kg, and an average BMI of 20.6 ± 1.7 kg/m^2^. Adherence to the intervention sessions, consisting of SSG, was 95.6% for the freeD group and 96.1% for the limitedD group.

The study protocol was initially authorized by the Ethics Committee of the Chengdu Institute of Physical Education (approval code 161/2024). Informed consent was also obtained from all participants, as well as their legal guardians. Ethical guidelines set forth in the Declaration of Helsinki for research involving human subjects were followed throughout the study.

### 2.2 Study design

A randomized controlled design was used in the study, incorporating two experimental intervention groups (freeD and limitedD) alongside the standard in-court basketball training, with a control group engaging only in regular in-court basketball training. Participants were recruited from four local basketball teams through convenience sampling.

To minimize the impact of specific club training routines on the outcomes, players from each team were randomly assigned to one of three groups, ensuring balanced participant numbers within each team. The random assignment was conducted using opaque envelopes and a 1:1 ratio, assigning players to groups prior to the initial assessment. This process ensured equal chances of group placement and maintained allocation concealment.

Evaluators, who were independent of the research team and unaware of group assignments and training interventions, conducted assessments 1 week before the intervention and immediately after the eighth week. However, due to logistical constraints in the training process, neither the players nor the researchers administering the protocols were blinded to the group assignments.

### 2.3 Interventions

The study used SSG interventions as an extra-time program that supplemented regular in-court basketball training sessions. The remaining in-court sessions were exclusively managed by the basketball coaches, while the researchers focused solely on implementing the experimental interventions. Over an 8-week period, the experimental groups participated in two additional SSG sessions weekly, while the control group engaged only in standard in-court sessions. The first session was held 48 h after a match, and the second session occurred 72 h later. Conducted before the regular training, these sessions started with a standardized warm-up that included 5 min of jogging, 10 min of dynamic stretching for the upper and lower limbs, 5 min of jumping exercises, and 5 min of individual technical actions. Training plans for the sessions during the intervention period are presented in Table 1.

**TABLE 1:** Detailed SSGs training program.

Week	freeD | S1	freeD | S2	limitedD | S1	limitedD | S2
W1	3v3 game with baskets on both sides. Court: 15 × 7 m. Free play 4 sets of 3 min each, with 2 min rest between sets	3v3 game with ball possession, awarding one point for every 10 consecutive passes. Court: 15 × 7 m. Free play 4 sets of 3 min each, with 2 min rest between sets	3v3 game with baskets on both sides. Court: 15 × 7 m. Dribbling while moving forward was not allowed 4 sets of 3 min each, with 2 min rest between sets	3v3 game with ball possession, awarding one point for every 10 consecutive passes. Court: 15 × 7 m. Dribbling while moving forward was not allowed 4 sets of 3 min each, with 2 min rest between sets
W2	2v2 game with baskets on both sides. Court: 15 × 7 m. Free play 6 sets of 2 min each, with 2 min rest between sets	2v2 game with ball possession, awarding one point for every 6 consecutive passes. Court: 15 × 7 m. Free play 6 sets of 2 min each, with 2 min rest between sets	2v2 game with baskets on both sides. Court: 15 × 7 m. Dribbling while moving forward was not allowed 6 sets of 2 min each, with 2 min rest between sets	2v2 game with ball possession, awarding one point for every 6 consecutive passes. Court: 15 × 7 m. Dribbling while moving forward was not allowed 6 sets of 2 min each, with 2 min rest between sets
W3	3v3 game with baskets on both sides. Court: 15 × 7 m. Free play 4 sets of 3 min each, with 2 min rest between sets	3v3 game with ball possession, awarding one point for every 10 consecutive passes. Court: 15 × 7 m. Free play 4 sets of 3 min each, with 2 min rest between sets	3v3 game with baskets on both sides. Court: 15 × 7 m. Dribbling while moving forward was not allowed 4 sets of 3 min each, with 2 min rest between sets	3v3 game with ball possession, awarding one point for every 10 consecutive passes. Court: 15 × 7 m. Dribbling while moving forward was not allowed 4 sets of 3 min each, with 2 min rest between sets
W4	2v2 game with baskets on both sides. Court: 15 × 7 m. Free play 6 sets of 2 min each, with 2 min rest between sets	2v2 game with ball possession, awarding one point for every 6 consecutive passes. Court: 15 × 7 m. Free play 6 sets of 2 min each, with 2 min rest between sets	2v2 game with baskets on both sides. Court: 15 × 7 m. Dribbling while moving forward was not allowed 6 sets of 2 min each, with 2 min rest between sets	2v2 game with ball possession, awarding one point for every 6 consecutive passes. Court: 15 × 7 m. Dribbling while moving forward was not allowed 6 sets of 2 min each, with 2 min rest between sets
W5	3v3 game with baskets on both sides. Court: 15 × 7 m. Free play 5 sets of 3 min each, with 2 min rest between sets	3v3 game with ball possession, awarding one point for every 10 consecutive passes. Court: 15 × 7 m. Free play 5 sets of 3 min each, with 2 min rest between sets	3v3 game with baskets on both sides. Court: 15 × 7 m. Dribbling while moving forward was not allowed 5 sets of 3 min each, with 2 min rest between sets	3v3 game with ball possession, awarding one point for every 10 consecutive passes. Court: 15 × 7 m. Dribbling while moving forward was not allowed 5 sets of 3 min each, with 2 min rest between sets
W6	2v2 game with baskets on both sides. Court: 15 × 7 m. Free play 8 sets of 2 min each, with 2 min rest between sets	2v2 game with ball possession, awarding one point for every 6 consecutive passes. Court: 15 × 7 m. Free play 8 sets of 2 min each, with 2 min rest between sets	2v2 game with baskets on both sides. Court: 15 × 7 m. Dribbling while moving forward was not allowed 8 sets of 2 min each, with 2 min rest between sets	2v2 game with ball possession, awarding one point for every 6 consecutive passes. Court: 15 × 7 m. Dribbling while moving forward was not allowed 8 sets of 2 min each, with 2 min rest between sets
W7	3v3 game with baskets on both sides. Court: 15 × 7 m. Free play 5 sets of 3 min each, with 2 min rest between sets	3v3 game with ball possession, awarding one point for every 10 consecutive passes. Court: 15 × 7 m. Free play 5 sets of 3 min each, with 2 min rest between sets	3v3 game with baskets on both sides. Court: 15 × 7 m. Dribbling while moving forward was not allowed 5 sets of 3 min each, with 2 min rest between sets	3v3 game with ball possession, awarding one point for every 10 consecutive passes. Court: 15 × 7 m. Dribbling while moving forward was not allowed 5 sets of 3 min each, with 2 min rest between sets
W8	2v2 game with baskets on both sides. Court: 15 × 7 m. Free play 8 sets of 2 min each, with 2 min rest between sets	2v2 game with ball possession, awarding one point for every 6 consecutive passes. Court: 15 × 7 m. Free play 8 sets of 2 min each, with 2 min rest between sets	2v2 game with baskets on both sides. Court: 15 × 7 m. Dribbling while moving forward was not allowed 8 sets of 2 min each, with 2 min rest between sets	2v2 game with ball possession, awarding one point for every 6 consecutive passes. Court: 15 × 7 m. Dribbling while moving forward was not allowed 8 sets of 2 min each, with 2 min rest between sets

W, week; S, session; freeD, small-sided games in free play; limitedD, small-sided games with limited dribbling.

In games with baskets, rebounds were allowed immediately after a shot, but three-point shots were prohibited. The freeD group was permitted to play freely without any restrictions on dribbling. In contrast, the limitedD group was not allowed to advance by dribbling. Instead, they were required to identify teammates, make a pass, and then move to create subsequent passing lanes and opportunities for progression. To ensure a competitive atmosphere, the intervention sessions involved teams playing against various opponents during repetitions, with each game’s results contributing to their overall points. Coaches balanced the teams based on players’ proficiency, physical attributes, and playing positions, maintaining consistent team compositions throughout the study. Tactical and strategic guidance, along with verbal encouragement, was avoided by coaches during game sessions. Balls were strategically placed closer to the baskets to facilitate faster repositioning.

### 2.4 Assessments and outcomes

The evaluations took place twice: once in the week prior to the intervention and again in the week following it. To ensure uniformity, the assessments were scheduled on the same days of the week, precisely 48 h after the last match. These evaluations were conducted indoors (private room and court) in a controlled environment, set at 21°C with a relative humidity of 50%, and took place during the afternoon hours.

Each evaluation followed the same protocol. First, demographic information was collected, and anthropometric measurements were taken. Then, the participants performed a standardized warm-up routine under the supervision of the evaluation team, which included 5 min of jogging, 10 min of dynamic stretching for both the upper and lower limbs, 5 min of jumping exercises, and 5 min of individual technical actions. Following the warm-up, participants completed the following tests in the same order: (i) 30-s Wingate test; and (ii) the Yo-Yo Intermittent Recovery test level 1.

#### 2.4.1 Anthropometric measurements

To ensure accuracy and consistency, a standardized procedure was followed by two assessors, both highly experienced in anthropometric assessments. Each assessor, with over 3 years of relevant experience and certified in physical education and sports sciences, had completed specialized training workshops. Participants were asked to wear a T-shirt, shorts, and no socks during the measurements.

For height measurements, participants stood with their backs against the height scale, looking forward to align the Frankfort Plane. The assessor then adjusted the stadiometer marker (ADE MZ10042, ADE, Germany) positioned in front of them. Next, body mass was measured with participants standing upright on an electronic flat scale (SECA Model 813, Germany), facing forward. The BMI was calculated using the formula: body mass in kilograms divided by the square of height in meters.

#### 2.4.2 The 30-s Wingate test

To measure the peak power output (PPO) and average power output (APO), a 30-s maximal Wingate test was conducted. Using a mechanically braked cycle ergometer (model 894E, Monark, Sweden), participants pedaled against a resistance set to 0.075 kg kg^–1^ of their body mass. The test began with participants pedaling as fast as they could against the device’s inertial resistance, after which the individualized load was applied. Verbal encouragement was given to athletes throughout the test to pedal at maximum speed for the entire 30 s. The maximal power reached at the 5-s mark, along with the average power over the duration of the test, were recorded as PPO (W) and APO (W), respectively.

#### 2.4.3 The Yo-Yo intermittent recovery test–Level 1

The Yo-Yo Intermittent Recovery Test–Level 1 (YYIRT) was used to assess the players’ aerobic capacity. Previously tested as a basketball-specific test, it was designed as a valid test to evaluate both aerobic fitness and game-related endurance (Castagna et al., 2008). The test consisted of repeated 20-m shuttle runs, with intensity progressively increasing as the test advanced. It began with an initial speed of 8 km/h, requiring participants to run back and forth between two markers 20 m apart, with a 10-s recovery period after each shuttle. The pace was dictated by audio beeps emitted from a recording system. The time between beeps started at a pace of 8 km/h and progressively decreased, increasing the required running speed with each level. The running speed increased by 0.5 km/h with each new level, continuing until the participant could no longer meet the required pace. The test concluded when the player could no longer complete a shuttle in time or missed two consecutive shuttles, and the distance covered (measured in meters) was recorded as the outcome.

### 2.5 Statistical methods

Before conducting inferential analyses, the normal distribution of the sample was assessed using the Kolmogorov-Smirnov test (p > 0.05). Levene’s test was used to verify the homogeneity of variance assumptions (p > 0.05). A mixed ANOVA (time: baseline and post-intervention * group: free, limited, and control) was conducted, with partial eta squared ( $\eta_{p}^{2}$ ) used to evaluate effect sizes. These were interpreted based on established thresholds (Richardson, 2011): >0.01 (small), >0.06 (moderate), and >0.14 (large). Post-hoc comparisons were carried out using the Bonferroni test. Additionally, Cohen’s standardized effect size (*d*) was used to determine the magnitude of difference in pairwise comparisons, with magnitudes interpreted as follows (Hopkins et al., 2009): 0.0–0.2, trivial; 0.2–0.6, small; 0.6–1.2, moderate; 1.2–2.0, large. Statistical analyses were performed using JASP software (version 0.18.3, University of Amsterdam, Netherlands), with a significance level set at *p* < 0.05.

## 3 Results

The Mixed ANOVA revealed significant interactions between groups and time for YYIRT (*F* = 230.368; *p* < 0.001; $\eta_{p}^{2}$ = 0.915), PPO (*F* = 190.357; *p* < 0.001; $\eta_{p}^{2}$ = 0.899), and APO (*F* = 317.715; *p* < 0.001; $\eta_{p}^{2}$ = 0.937).

Table 2 presents the descriptive statistics for each group’s outcomes at baseline and post-intervention. Comparisons of YYIRT between groups at post-intervention revealed that limitedD performed significantly better than both freeD (*p* = 0.035; *d* = 1.038, moderate effect size) and the control group (*p* < 0.001; *d* = 2.050, large effect size), while freeD also showed significantly better performance (*p* = 0.021; *d* = 0.082, trivial effect size) than the control group. Regarding PPO at post-intervention, limitedD was significantly better than the control group (*p* = 0.043; *d* = 0.943, moderate effect size). Finally, for APO at post-intervention, limitedD was significantly better than both freeD (*p* = 0.043; *d* = 0.928, moderate effect size) and the control group (*p* < 0.001; *d* = 1.793, moderate effect size), while freeD also exhibited significantly better performance than the control group (*p* = 0.046; *d* = 0.036, trivial effect size).

**TABLE 2:** Descriptive statistics (mean ± standard deviation) for each group’s outcomes at baseline and post-intervention.

	freeD (n = 15)	limitedD (n = 15)	Control (n = 16)	Between-group analysis	Pairwise comparisons
YYIRT (m)					
Pre	1465.3 ± 120.6	1488.0 ± 114.3	1451.3 ± 155.4	*F*_*2,43*_ = 0.304; *p* = 0.739; $\eta_{p}^{2}$ = 0.014	freeD ≈ limitedD (*p >* 0.999) freeD ≈ control (*p >* 0.999) limitedD ≈ control (*p >* 0.999)
Post	1570.7 ± 124.6*	1686.7 ± 99.0*	1447.5 ± 134.4	*F*_*2,43*_ = 15.231; *p* < 0.001; $\eta_{p}^{2}$ = 0.415	#freeD < limitedD (*p* = 0.035) #freeD > control (*p* = 0.021) #limitedD > control (*p* < 0.001)
PPO (W)					
Pre	700.9 ± 31.4	703.3 ± 31.7	696.8 ± 27.2	*F*_*2,43*_ = 0.186; *p* = 0.831; $\eta_{p}^{2}$ = 0.009	freeD ≈ limitedD (*p >* 0.999) freeD ≈ control (*p >* 0.999) limitedD ≈ control (*p >* 0.999)
Post	712.1 ± 31.4*	723.7 ± 30.1*	696.4 ± 27.8	*F*_*2,43*_ = 3.288; *p* = 0.047; $\eta_{p}^{2}$ = 0.133	freeD ≈ limitedD (*p* = 0.876) freeD ≈ control (*p* = 0.449) #limitedD > control (*p =* 0.043)
APO (W)					
Pre	438.3 ± 17.2	434.9 ± 17.8	435.2 ± 16.4	*F*_*2,43*_ = 0.185; *p* = 0.832; $\eta_{p}^{2}$ = 0.009	freeD ≈ limitedD (*p >* 0.999) freeD ≈ control (*p >* 0.999) limitedD ≈ control (*p >* 0.999)
Post	451.4 ± 16.5*	467.6 ± 18.4*	435.6 ± 17.3	*F*_*2,43*_ = 13.069; *p* < 0.001; $\eta_{p}^{2}$ = 0.378	#freeD < limitedD (*p* = 0.043) #freeD > control (*p* = 0.046) #limitedD > control (*p* < 0.001)

YYIRT, Yo-Yo Intermittent Recovery Test–Level 1; PPO, peak power output; APO, average power output; freeD, small-sided games in free play; limitedD, small-sided games with limited dribbling; *: significantly different (*p* < 0.05) within group; ≈: approximately similar; #: significantly (*p* < 0.05) different between groups; <: significantly smaller; >; significantly greater.

**FIGURE 2:**
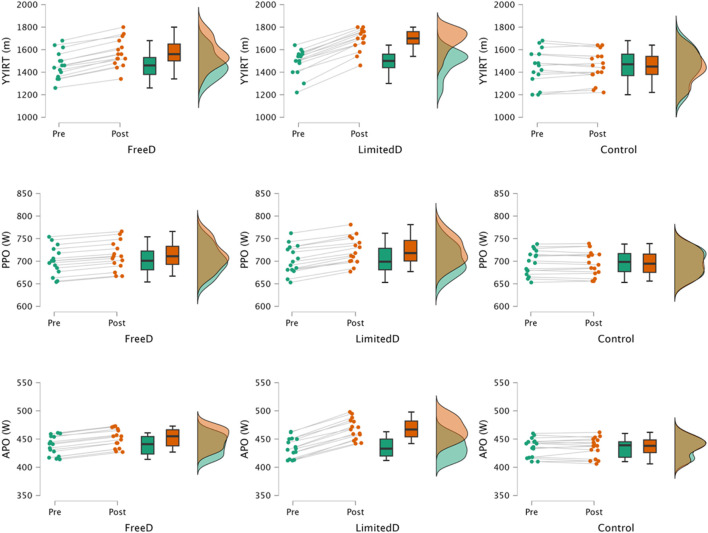
Raincloud plots of the Yo-Yo Intermittent Recovery Test (YYIRT), peak power output (PPO), and average power output (APO) for the groups: small-sided games in free play (freed), small-sided games with limited dribbling (limitedD), and control.

Figure 2 illustrates the data for both pre- and post-intervention. In the freeD group, YYIRT (*p* < 0.001; *d* = 0.860, moderate effect size), PPO (*p* < 0.001; *d* = 0.357, small effect size), and APO (*p* < 0.001; *d* = 0.777, moderate effect size) all showed significant improvement from baseline to post-intervention. Similarly, the limitedD group revealed significant improvements in YYIRT (*p* < 0.001; *d* = 1.863, large effect size), PPO (*p* < 0.001; *d* = 0.660, moderate effect size), and APO (*p* < 0.001; *d* = 1.807, large effect size) from baseline to post-intervention. The control group did not significantly enhance any of the measures, including YYIRT (*p* = 0.571; *d* = 0.026, trivial effect size), PPO (*p* = 0.617; *d* = 0.015, trivial effect size), and APO (*p* = 0.627; *d* = 0.024, trivial effect size).

## 4 Discussion

The research found that limitedD was significantly more effective than freeD in enhancing YYIRT and APO. Furthermore, the limitedD group was the only one to show a significant improvement in PPO compared to the control group.

Our results revealed that both experimental groups showed significant improvements in aerobic capacity and were significantly better than the control group. However, it was also observed that the limitedD group exhibited significantly greater improvements than the freeD group. Although no comparative experimental studies have specifically investigated this topic, our results may be supported by previous research (Conte et al., 2015; Ferioli et al., 2020) that has shown that limiting dribbling in basketball during SSGs generally increases physiological and locomotor demands. Limiting dribbling likely led to a greater emphasis on movement and positioning, such as more frequent off-ball runs, faster transitions, and more intense efforts to support teammates (Ferioli et al., 2020). This may have increased the overall metabolic cost of the activity, raising its intensity (Conte et al., 2015). The higher intensity, as evidenced by previous studies, likely contributed to greater cardiovascular strain and metabolic adaptations, including improved maximal oxygen uptake and lactate threshold (Delextrat and Martinez, 2014). Additionally, freeD was significantly more effective than the control group. Although it may not be as intense as limitedD, the introduction of SSGs proves effective in enhancing aerobic capacity due to their high-intensity and intermittent nature (Delextrat and Martinez, 2014; Vaquera et al., 2018; Clemente et al., 2021). This characteristic makes them suitable for taxing aerobic power throughout the drills, ultimately leading to long-term improvements in aerobic capacity (Delextrat and Martinez, 2014).

The current research also found that limitedD was the only experimental group that showed a significant improvement in peak power output (PPO) after the intervention compared to the control group, while no differences were observed between freeD and the control group. Limiting dribbling likely increased the intensity of physical effort, as players must rely more on aerobic and anaerobic energy systems to move quickly, change direction, and create offensive opportunities without the benefit of dribbling (Ferioli et al., 2020). The increased locomotor demands may have stimulated greater neuromuscular adaptations, including improved muscular power, which could contribute to enhanced performance in anaerobic power (Cao et al., 2024). Specifically, repeated high-intensity efforts during the limited dribbling conditions may have led to greater recruitment of fast-twitch muscle fibers, driven by the need for more frequent accelerations and decelerations, potentially enhancing their capacity for rapid energy production (Xu et al., 2024). In contrast, the freeD group, which allowed free dribbling, may have involved more variable intensity and less consistent engagement of the anaerobic systems, which could explain the lack of improvement in peak power output observed in this group.

Regarding the average power output (APO), both experimental groups showed significant improvements compared to the control group after the intervention. However, it was also observed that limitedD was significantly better than freeD. Restricting dribbling possibly increased the intensity of the game by forcing players to rely more on their aerobic and anaerobic energy systems to maintain movement, create space, and support team dynamics without the option of frequently stopping to dribble (Conte et al., 2015; Ferioli et al., 2020). This increased intensity likely led to greater engagement of both the aerobic and anaerobic systems, enhancing muscle endurance, energy production, and efficiency. Although the freeD group was effective, it allowed for dribbling, which may have reduced the overall physical demand (Conte et al., 2015; Ferioli et al., 2020) and the balance between aerobic and anaerobic effort, resulting in less pronounced improvements in average power output.

LimitedD likely increased locomotor demands by requiring more off-the-ball movement and positional adjustments (Conte et al., 2015; Ferioli et al., 2020), potentially leading to greater engagement of both the aerobic and anaerobic energy systems compared to freeD. The continuous activity at moderate-to-high intensities may have enhanced aerobic capacity, stimulating adaptations such as increased mitochondrial density and capillary growth (Delextrat and Martinez, 2014). Simultaneously, the frequent transitions and high-intensity efforts needed for rapid repositioning and quick passes could have boosted anaerobic performance, improving phosphocreatine resynthesis and lactate tolerance (Conte et al., 2015; Ferioli et al., 2020). This dual enhancement was possibly due to the elevated overall physical demands imposed by the constraint, promoting a broader spectrum of metabolic adaptations than freeD.

Despite the interesting findings in this study, some limitations should be considered. The age group and sex of the participants may have limited the generalizability of the results to broader populations, such as elite athletes, adults, or women. Additionally, physiological and locomotor demands during each session were not monitored, which could have provided a better understanding of the relationship between the physical efforts experienced and the observed outcomes. Future research should aim to expand on these findings by including larger, more diverse sample sizes and investigating the connection between the actual efforts experienced during sessions and the outcomes observed. Finally, a potential limitation of this study is the impact that performing high-intensity limitedD prior to regular training sessions may have on subsequent training, influencing physical readiness, fatigue levels, and skill execution. Future research should explore the optimal timing and sequencing of such interventions within training cycles to better understand their effects on overall performance and recovery.

Despite the limitations, the results of this study suggest that limitedD can be particularly effective in intensifying the game and leading to greater improvements in both aerobic and anaerobic performance. This makes it a potentially valuable strategy for designing SSGs in basketball training. Coaches may consider incorporating limited-dribbling conditions into SSGs to boost the intensity of training sessions, improve cardiovascular endurance, and enhance anaerobic power. Specifically, limiting dribbling could be strategically used during pre-season to improve players’ physical conditioning or during skill-development phases to focus on enhancing off-ball movement and team dynamics. Additionally, this approach can be beneficial for targeting anaerobic endurance and fostering tactical awareness in youth players. Coaches should integrate these sessions into broader training programs, ensuring appropriate sequencing to maximize performance gains without compromising recovery or skill execution.

## 5 Conclusion

In conclusion, the findings of this study highlight the effectiveness of limitedD in promoting both aerobic and anaerobic adaptations compared to freeD. While both experimental groups showed significant improvements in aerobic capacity and APO relative to the control group, limitedD was better than freeD in enhancing YYIRT and APO, and was the only group to show a significant improvement in PPO. Although some limitations, the study suggests that limiting dribbling during SSGs is a promising strategy for increasing SSG design. Integrating limited-dribbling conditions into SSG can improve training intensity, enhance cardiovascular endurance, and improve anaerobic power, proving especially beneficial during pre-season or skill-building phases. Coaches should incorporate these sessions into ample training plans, ensuring appropriate sequencing to maximize performance gains while supporting recovery and skill development.

## Data Availability

The raw data supporting the conclusions of this article will be made available by the authors, without undue reservation.

## Abbreviations

APO, average power output; PPO, peak power output; freeD, small-sided games with free play; limitedD, small-sided games with limited dribbling; YYIRT, Yo-Yo Intermittent Recovery Test–Level 1.
